# 

**Published:** 1817-10

**Authors:** 


### CATALOGUE OF MEDICAL BOOKS.

CASES of Diseased Prepuce and Scrotum, illustrated by Etchings. By William Wadd, Esq. Surgeon Extraordinary to his Royal Highness the Prince Regent.

Sketch of the History and Cure of Febrile Diseases, more particularly as they appear in the West Indies, among the Soldiers of the British Army. By Robert Jackson, M.D.

History of the Practice of Vaccination. By John Moore, Director of the National Vaccine Establishment, Surgeon of the 2d Reg. of Guards, and Member of the Royal College of Surgeons in London.

Chemical Amusement, comprising a Series of curious and instructive Experiments in Chemistry, which are easily performed, and unattended by danger. By Frederick Accum, Operative Chemist. 7s.

The Naturalist's Pocket-Book, or Tourist's Companion; being a brief Introduction to the different branches of Natural History, with approved Methods for collecting and preserving Quadrupeds, Birds, Reptiles, Fishes, Insects, Shells, Corals, Seeds, Plants, Woods, Fossils, Minerals, &c. By George Graves, F.L.S. 1 vol. 8vo. with 8 plates; price 14s. plain, 21s. coloured.

No. 24 of the new edition of Curtis's Flora Londinensis. By G. Graves, F.L.S. In royal folio, with 6 plates; price 10s. plain, or 16s. coloured.

A Catalogue of Medical Books now selling by Highley and Son, Medical Booksellers, 174, Fleet-street, containing the most modern and approved Works on Anatomy, Medicine, Surgery, Materia Medica, Chemistry, &c. &c. corrected to the present time: to which is added, a List of all the Lectures delivered in London, with the terms, hours of attendance, &c. &c.

A Catalogue and Appendix of Books in Anatomy, Medicine, Surgery, Midwifery, Chemistry, Botany, &c. new and second-hand, sold by Anderson and Chase, Medical Booksellers, 40, West Smithfield: to which is added, a complete List of the Lectures delivered in London, with their terms, hours of attendance, &c.

An Essay on the Chemical History and Medical Treatment of Urinary Calculi; with plates. By Alexander Marcet, M.D. F.R.S.

Observations on the Casual and Periodical Influence of the particular States of the Atmosphere on Human Health and Diseases, particularly Insanity; with a table of reference to authors. By Thomas Forster, F.L.S. &c.

---

### *NOTICES TO CORRESPONDENTS.*

*The extract from the Moniteur (Public Utility) can only be admitted as an advertisement on the Cover.*

*We are much obliged to our correspondent for the favour concerning Nitrous Acid; but, under the present mode of conducting the Journal, we cannot admit histories of venereal sores, without a more minute description of their appearance. We must therefore wait for his further communication.*

PROPHYLAX, *and several others, will appear, if possible, in our next.—Does our correspondent at Berwick wish his name to be added to his favour?*

*We feel professionally for our Bristol correspondent; but, as the cause is scarcely known, excepting in his own neighbourhood, we would advise him to print the affidavit, either in the Bristol papers, or to circulate it among those who are likely to interest themselves in the question.*

